# Association of miR-34a and miR-143 levels with PPARγ gene expression in adipose tissues of non-diabetic adults

**DOI:** 10.1186/s40101-022-00286-0

**Published:** 2022-04-09

**Authors:** Maryam Zarkesh, Kimia Tabaei, Mahdi Akbarzadeh, Afsoon Daneshafrooz, Azita Zadeh-Vakili

**Affiliations:** 1 Cellular and Molecular Endocrine Research Center, Research Institute for Endocrine Sciences, Shahid Beheshti University of Medical Sciences, Tehran, Iran; 2 Endocrine Research Center, Research Institute for Endocrine Sciences, Shahid Beheshti University of Medical Sciences, Tehran, Iran

**Keywords:** PPARγ expression, MicroRNAs, Adipose tissue, Non-diabetic, Obesity, Adults

## Abstract

**Background:**

Peroxisome proliferator-activated receptor gamma (PPARγ) is a promising therapeutic molecule. Epigenetic mechanisms, including non-coding RNAs, regulate the expression level of the PPARγ gene.

**Objective:**

We aimed to examine the PPARγ expression in non-diabetic individuals in four body mass index (BMI) categories and its association with miR-34a and miR-143 expression.

**Methods:**

Visceral and subcutaneous adipose tissues (VAT and SAT) samples were collected from patients undergoing bariatric or elective open abdominal surgeries. The subjects (mean age: 42±14.8 years) included 18 normal-weight, 19 overweight, 18 obese, and 19 morbidly obese individuals. The RNAs levels were determined by quantitative real-time PCR.

**Results:**

The PPARγ expression was significantly upregulated in both adipose depots of the morbidly obese subjects compared to the normal group. SAT PPARγ level was significantly increased in the obese group compared to the normal-weight group (*P*<0.01); this increase was also significant in the SAT of morbidly obese subjects compared to the overweight cases (*P*=0.02). Differences in the regulation of PPARγ expression in both SAT and VAT were significant between the four groups (*P*<0.05). While miR-143 was overexpressed in the SAT of obese and morbidly obese individuals compared to the normal-weight group, the pairwise comparison showed no significant difference in the miR-34a expression of SAT between the four BMI groups (*P*>0.01). After controlling for the confounding factors, the expression of VAT PPARγ was directly associated with the miR-34a level in the normal-weight group (*β*=0.311, *P*=0.010). A negative association was observed between the VAT PPARγ expression and miR-34a expression in obese cases (*β* = − 0.594, *P*=0.039).

**Conclusion:**

The results also confirmed the regulatory function of microRNAs in the PPARγ expression and adipogenesis.

## Introduction

According to the World Health Organization (WHO) reports on obesity and overweight, the prevalence of obesity has nearly tripled since 1975 worldwide [1]. Statistics show that in 2016, 39% of adults aged 18 years or above were overweight, while 13% were obese. The rapidly increasing prevalence of obesity has made it a significant public health crisis. Therefore, developing effective strategies and novel interventions for preventing and treating obesity is urgently needed. Also, understanding the molecular mechanisms underlying the etiology of obesity, which are associated with obesity comorbidities, may help researchers develop feasible approaches for obesity prevention. 

Peroxisome proliferator-activated receptor gamma (PPARγ) is a ligand-activated transcription factor of the nuclear hormone receptor superfamily, which plays a vital role in the differentiation of adipocytes [2]. It has been reported that ectopic expression and activation of PPARγ in a fibroblast cell line results in its differentiation into adipocytes [3]. Therefore, this molecule has been considered a candidate gene, which may be involved in obesity development; therefore, it may be a therapeutic target for obesity treatment [4].

Nevertheless, the relationship between the PPARγ expression pattern in different adipose tissues and obesity is unclear. So far, the inclusion of subjects in different body mass index (BMI) categories and differences in the inclusion criteria have yielded inconsistent results. For example, Bortolotto et al. reported no significant difference in the expression of PPARγ in the subcutaneous adipose tissue (SAT) of morbidly obese subjects (mean BMI = 50.3±2.3 kg/m^2^) compared to the lean group [5]. On the other hand, Rodríguez-Acebes et al. reported the decreased expression of PPARγ in morbidly obese subjects (BMI >35 kg/m^2^) [6], while Ruschke et al. reported a significant increase in obese individuals (BMI >30 kg/m^2^) [7].

Since PPARγ is a transcription factor involved in different biological processes, its gene expression is regulated by genetic and epigenetic regulation, including non-coding RNAs [8]. Microribonucleic acids (microRNAs), a group of non-coding RNAs with 19-25 nucleotides, are considered epigenetic modulators. They can bind to the 3′-untranslated region of target messenger RNAs and subsequently suppress protein translation. According to our literature review, recent studies have shown the role of microRNAs, including miR-143 and miR-34a, in fat cell development and obesity [9, 10]; however, their functional role in obesity remains unknown. The miR-143 affects human adipocytes in cultures and high-fat-diet-fed mice; its expression correlates with the PPARγ expression [11]. Ablation of miR-34a in mice fed a high-fat diet decreased the expression of PPARγ. Both of these microRNAs showed a progressively increasing expression during the development of obesity in mice [12].

Despite the importance of PPARγ as a promising therapeutic molecule, there are inconsistent results regarding its expression in the human adipose tissues. On the other hand, that previous studies on miR-143 and miR-34a molecules have been performed either in animals [11, 13] or human tissues other than adipose tissue [14]; and there is no research on the correlation of miR-143 and miR-34a levels with PPARγ expression in the human adipose tissues. Therefore, we performed this study on non-diabetic individuals in four categories of BMI. We aimed to determine the correlation between the expression of PPARγ and the expression of miR-143 and miR-34a as possible modulators of PPARγ, which can be used in new obesity treatment.

## Methods

### Subjects

In this descriptive, case-control study, 74 subjects were selected among patients undergoing general (e.g., gallbladder and appendix surgeries) or obesity surgeries at Seyed Mostafa Khomeini and Khatam-al-Anbya hospitals in Tehran, Iran. The inclusion criteria were as follows: age ≥19 years; lack of type 2 diabetes (T2D); no pregnancy; and lack of any cancer. This study was conducted according to the Declaration of Helsinki and the RIES institutional guidelines. Written informed consent was also obtained from all participants.

### Anthropometric and laboratory measurements

Weight, height, and waist, hip, wrist, and neck circumferences were measured according to the standard protocols, as described previously [15, 16]. Body mass index (BMI) was calculated as weight (kg) divided by square of height (m^2^). Systolic blood pressure (SBP) and diastolic blood pressure (DBP) were recorded using a standard sphygmomanometer after at least ten minutes of rest [17]. Blood samples were collected from all participants after 10-12 hours of overnight fasting. Fasting plasma glucose (FPG), triglyceride (TG), and total cholesterol (TC) levels were also measured using commercial kits (Pars Azmoon Inc., Tehran, Iran). Insulin was measured using the enzyme-linked immunosorbent assay (ELISA) with a Mercodia ELISA kit (Uppsala, Sweden). Inter-and intra-assay coefficients of variation (CVs) for FPG, TG, TC, and insulin were <5%. Finally, the subjects were divided into four groups, based on BMI: 18 normal weight (BMI <25 kg/m^2^), 19 overweight (BMI = 25–30 kg/m^2^), 18 obese (BMI = 30–40 kg/m^2^), and 19 morbidly obese (BMI≥40 kg/m^2^) individuals.

### Physical activity and dietary measurements

Physical activity was assessed using a validated and reliable Persian version of the International Physical Activity Questionnaire Long-Form (IPAQ-LF); the metabolic equivalent minutes (MET) per week was also calculated [18, 19]. Dietary intakes were collected through face-to-face interviews by a trained dietitian, using a valid and reliable semi-quantitative food frequency questionnaire (FFQ) [20, 21].

### Adipose tissue sample collection

To determine the expression of PPARγ and microRNAs, 50 mg of SAT and 30 mg of VAT were collected in RNase and DNase-free microtubes, frozen immediately in liquid nitrogen, and then kept at a temperature of -80°C.

### RNA extraction and quantitative real-time reverse transcription-polymerase chain reaction (qRT-PCR)

For total RNA extraction using the TRIzol reagent (Invitrogen, USA, Cat. No.: 15596-026), tissues samples were homogenized (MM400 homogenizer, Retsch, Germany), and total RNA was extracted, according to the manufacturer's recommendations. The quality of extracted RNA was measured by determining the absorbance ratio at 260 and 280 nm (A_*260*/*280*_). Total complementary DNA (cDNA) was synthesized using the RevertAid^TM^ First-Strand cDNA Synthesis Kit (Thermo Fisher Scientific, USA). Also, microRNA cDNA was generated by a microRNA cDNA synthesis kit (ParsGenome, Iran). The primer sequences were as follows: PPARγ (forward): 5′-CCT CAT GAA GAG CCT TCC AAC-3′ and PPARγ (reverse): 5′-ACC CTA GCA TCC TTC ACA AGC-3′; GAPDH (forward): 5′-CTG CTC CTC CTG TTC GAC AGT-3′ and GAPDH (reverse): 5′-CCG TTG ACT CCG ACC TTC AC-3′.

The qRT-PCR assay was performed using the SYBR Green PCR Master Mix (BioFact, Korea) and microRNAs (ParsGenome, Iran) in a Corbett Rotor-Gene 6000 machine (Sydney, Australia). GAPDH [22] and U6 snRNA were used as internal controls to normalize the mRNA and miR expression levels, respectively. The following cycling program was used in this study: five minutes at 95 °C, followed by 40 cycles at 95 °C for 5 s, at 62 °C for 20 s, and at 72 °C for 30 s. Duplicate reactions were performed for each sample.

### Statistical analysis

The Kolmogorov-Smirnov test assessed the normal distribution of data. Normally distributed variables were reported as mean±SD. One-way analysis of variance (ANOVA) was used to determine significant differences in the mean values of variables between the four groups of BMI. ANCOVA test was also used to adjust for age and sex as confounding factors. Moreover, the relative gene expression was calculated by the 2^-ΔΔCT^ method [23]. Kruskal-Wallis test was used to compare the expression levels between the four study groups. To examine differences between every two groups, Mann-Whitney U test with Bonferroni correction was performed (*P*<0.01 was considered significant).

Spearman’s correlation coefficients were determined for PPARγ, miR-143, and miR-34a expression in the SAT and VAT. A linear regression analysis was performed to determine the association of PPARγ level with miR-34a and miR-143 expression in the VAT and SAT. Age, sex, physical activity, and energy intake (kcal) were considered confounding variables and adjusted in the model. Data were analyzed using R.1.1, and statistical differences were considered significant at *P* value <0.05.

## Results

The clinical characteristics of all participants in the four studied groups are presented in Table 1. Of 74 subjects with a mean age of 42±14.8 years, 75% were women. Anthropometric variables, including BMI, waist, hip, wrist, and neck circumferences, age, and TC level, were significantly different between the normal-weight, overweight, obese, and morbidly obese groups (*P*<0.05); however, the SBP, DBP, TG, FPG, and insulin levels were not significantly different, even after controlling for age and sex.

**Table 1:** General anthropometric and biochemical information of the participants based on their obesity status

Parameter	Total (*n*=74)	Normal (*n*=18)	Overweight (*n*=19)	Obese (*n*=18)	Morbidly obese (*n*=19)	*p* ANOVA	*P* ANCOVA^c^
Age (year)	42.0±14.8^a^	42.5±16.3	49.4±15.3	41.7±14	34.4±10	0.017	-
Sex							
Male	18(25)^b^	5(27.8)	7(38.9)	1(5.50)	5(27.8)	0.161	-
Female	56(75)	13(23.2)	12(21.4)	17(30.4)	14(25)		
BMI (kg/m^2)^	32.8±9.7	22.2±1.8	27.7±1.3	35±3.2	46.5±4.7	<0.001	<0.001
WC (cm)	104±21.4	82±10.1	95.8±7.7	105.8±12.5	132.2±10.4	<0.001	<0.001
Hip circumference (cm)	114.2±22.7	90.3±13.1	104.2±7.2	112.1±16.3	141±12.1	<0.001	<0.001
Wrist circumference (cm)	17.7±1.6	16.6±1.3	17.4±1.2	17.1±1.3	19.1±1.3	<0.001	<0.001
Neck circumference (cm)	39±5.2	35.6±2	37.5±4.9	38.7±6.1	42.6±4.1	<0.001	<0.001
SBP (mmHg)	113±13.6	108±15.5	113±9.7	120±12.7	110±13.6	0.063	0.072
DBP (mmg)	72.3±9.1	70.7±8.2	71.0±8.9	75.3±9.3	71.8±10.0	0.454	0.333
TG (mg/dL)	91.6±61.5	88.3±72.6	69.5±11.8	107.7±60.1	103±75.7	0.285	0.274
FPG (mg/dL)	86.8±11.1	90.5±11.6	86.7±11.6	83.7±6.7	85.6±12.6	0.382	0.552
Insulin (IU/mL)	10.4±13	6.1±5.6	11.8±19.6	12.7±15	11.5±7.5	0.462	0.401
TC (mg/dL)	181.6±43.2	162.5±47.7	179±45.4	206.5±35.7	182.9±34.5	0.042	0.009

^a^ Mean±SD; ^b^ Number (%);^c^Adjusted for age and sex

*BMI* body mass index, *WC* waist circumference, *SBP* systolic blood pressure, *DBP* diastolic blood pressure, *TG* triglyceride, *FPG* fasting plasma glucose, *TC* total cholesterol, *Normal* BMI <25 kg/m^2^, *overweight* 25 kg/m^2^<BMI<30 kg/m^2^, *obese* 30 kg/m^2^<BMI<40 kg/m^2^, *morbidly obese* BMI≥40 kg/m^2^

### PPARγ expression

**Fig. 1:**
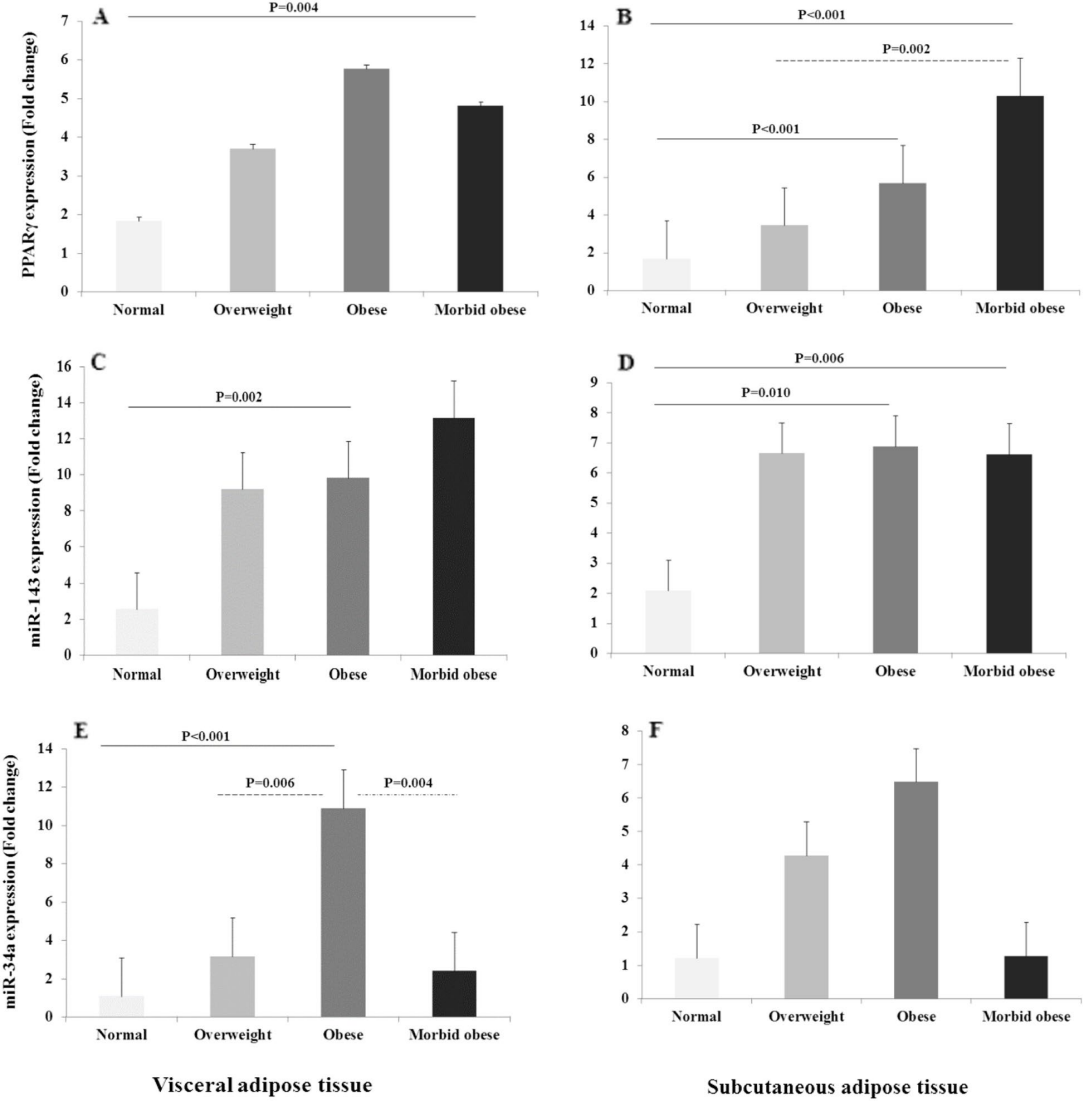
Comparison of PPARγ, miR-34a and miR-143 expression levels between different groups. The PPARγ mRNA levels in (**A**) VAT and (**B**) SAT of normal-weight, overweight, obese, and morbidly obese groups; miR-34a levels in (**C**) VAT and (**D**) SAT of normal-weight, overweight, obese, and morbidly obese groups; and miR-143 levels in (**E**) VAT and (**F**) SAT of normal-weight, overweight, obese, and morbidly obese groups. Kruskal–Wallis test was used to compare differences in expression between the four study groups (*P* value <0.05 was considered significant). To examine differences between every two groups, Mann-Whitney U test with Bonferroni correction was performed (*P* value <0.01 was considered significant)

The results of the Kruskal-Wallis test showed a significant difference in the PPARγ expression between the BMI groups (*P*=0.027 and *P*<0.001 for VAT and SAT, respectively). Mann-Whitney U test revealed that the VAT PPARγ levels were upregulated in morbidly obese subjects compared to the normal-weight group (*P*=0.004). Besides, the SAT PPARγ expression increased significantly in obese and morbidly obese individuals compared to the normal-weight ones (P<0.001). This increase was also significant in the SAT of morbidly obese individuals compared to overweight cases (P=0.02) (Fig. 1A, B).

### MiR-143 expression

Significant differences were observed in both adipose depots between the four BMI groups (*P*=0.021 and *P*=0.020 for VAT and SAT, respectively). After a pairwise comparison and Bonferroni correction, the miR-143 levels in both VAT and SAT were significantly upregulated in obese individuals compared to the normal-weight group (*P*=0.002 and *P*=0.010, respectively). Besides, there was a significant increase in the SAT miR-143 expression in morbidly obese subjects compared to the normal-weight group (*P*=0.006) (Fig. 1C, D).

### MiR-34a expression

The miR-34a was differentially expressed in the four BMI groups (P<0.001 and P=0.033 for VAT and SAT, respectively). After the pairwise comparison and Bonferroni correction, the VAT miR-34a expression was found to be higher in obese subjects compared to normal-weight, overweight, and morbidly obese subjects (*P*<0.001, *P*=0.006, and *P*=0.004, respectively). On the other hand, no significant differences were found between the four BMI groups regarding the SAT miR-34a level (*P*>0.05) (Fig. 1E, F).

### Correlations

**Fig. 2:**
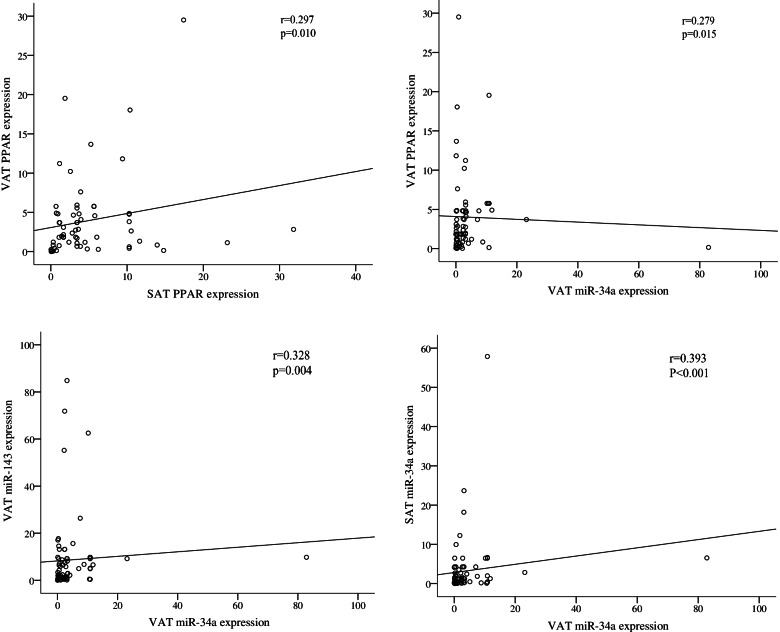
Spearman’s correlation coefficients for PPARγ, miR-143, and miR-34a expression in the SAT and VAT

In all participants, a positive correlation was found between the PPARγ expression in the VAT and SAT (*r*=0.297, *P*=0.010) and between the expression of PPARγ and miR-34a level in VAT (*r*=0.279, *P*=0.015). Besides, a significant positive correlation was found in miR-34a expression in the VAT and SAT (*r*=0.328, *P*=0.004). A significant positive correlation was also seen between miR-34a and miR-143 expression in the VAT (*r*=0.393, *P*<0.001) (Fig. 2).

### Linear regression analysis

To assess the association of PPARγ expression with miR-34a and miR-143 levels in the VAT and SAT of the four BMI groups, linear regression analyses were performed after adjustments for age, sex, physical activity, and energy intake (kcal); the results are presented in Table 2. After controlling for the confounding factors, the expression of VAT PPARγ was directly associated with the miR-34a level in normal subjects (*β*=0.311, *P*=0.010). Also, a negative association was observed between the VAT PPARγ expression and miR-34a level in obese subjects (*β*= − 0.594, *P*=0.039).

**Table 2:** Association of PPARγ expression with miR-34a and miR-143 levels

	Normal (***n***=19)				Overweight (***n***=19)				Obese (***n***=18)				Morbidly obese (***n***=19)			
	miR-34a		miR-143		miR-34a		miR-143		miR-34a	miR-143			miR-34a		miR-143	
	B standard	*P* value	B standard	*P* value	B standard	*P* value	B standard	*P* value	B standard	*P* value	B standard	*P* value	B standard	*P* value	B standard	*P* value
**VAT**																
Model 1	0.161	0.473	0.299	0.203	0.055	0.838	− 0.112	0.686	− 0.249	0.328	0.088	0.738	0.056	0.842	− 0.126	0.665
Model 2	0.311	0.010	0.089	0.535	− 0.028	0.914	− 0.177	0.506	− 0.594	0.039	0.239	0.433	− 0.004	0.988	− 0.205	0.514
**SAT**																
Model 1	− 0.473	0.108	0.034	0.896	− 0.136	0.583	0.017	0.943	− 0.469	0.097	− 0.096	0.699	0.453	0.147	− 0.175	0.519
Model 2	− 0.418	0.159	0.043	0.878	− 0.244	0.457	0.053	0.841	− 0.516	0.059	− 0.137	0.618	0.426	0.198	− 0.125	0.718

*VAT* visceral adipose tissues, *SAT* subcutaneous adipose tissue

Model 1: Adjusted for age and sex

Model 2: Adjusted for age, sex, physical activity, and energy intake (kcal)

## Discussion

We examined the expression level of PPARγ, as well as two related microRNAs in the SAT and VAT of overweight, obese, and morbidly obese adults. Expression levels of PPARγ in both adipose depots were significantly higher in morbid individuals than normal-weight ones. In SAT, overexpression was identified in the obese group compared to normal weight and between the morbidly obese and overweight group. We also found the significant overexpression of miR-34a in the VAT of obese subjects compared to the other groups. The miR-34a expression in the SAT of overweight and obese groups was significantly higher than the normal-weight group, while morbidly obese subjects showed no miR-34a overexpression. After controlling for confounding factors, the linear regression analysis in the normal-weight group showed that for a one-unit increase (fold change) in the miR-34a expression, there was an increase of 31% in the VAT PPARγ expression. On the contrary, a reduction of 51% in the VAT PPARγ expression was associated with a one-unit increase in the miR-34a expression in the obese group.

Many studies have reported different results regarding the expression of PPARγ in different adipose tissues of obese individuals. However, the use of different adipose tissues, differences in the BMI range for obesity definition, and also the inclusion of diabetic and non-diabetic cases have led to inconsistent results. Bortolotto et al. reported no differential PPARγ expression in the VAT of ten morbidly obese (mean BMI= 50.3±2.3 kg/m^2^) and ten non-obese (BMI range= 17.9–29.0 kg/m^2^) individuals. The mean FPG was in the normal range (77–153 mg/dL for the morbidly obese group). Based on their findings, the PPARγ mRNA expression increased significantly in the SAT of morbidly obese subjects [5].

Moreover, Ruschke et al. examined the expression of some candidate genes, including PPARγ, in the SAT of 58 obese (BMI>35 kg/m^2^; FPG: 5.9±1.4 mmol/L) and 58 lean (BMI<25 kg/m^2^; FPG: 5.4±2.9 mmol/L) individuals. According to their results, the expression of PPARγ significantly increased in obese subjects. In their study, the level of FPG in the obese group was significantly higher than the lean group (5.9±1.4 vs. 5.4±2.9 mmol/L) [24]. They reported no significant difference in the PPARγ expression in VAT of the other 37 obese subjects, who showed an insignificant difference in the FPG level (5.8±1 vs. 5.4±2.9 mmol/L).

Moreover, in a recent study by Castellano et al., BMI>40 kg/m^2^ was considered as morbid obesity [25]. They found no significant difference in the mRNA expression between the normoglycemic lean (*n*=10; glucose <100 mg/dL) and normoglycemic obese (*n*=10) individuals. Meanwhile, the PPARγ mRNA expression was significantly lower in the prediabetic obese group (*n*=9, glucose ≥100 to <125). These findings revealed that diabetes, rather than BMI, is a determinant of PPARγ expression in obese subjects. However, our approach in this study was somehow different. We only included non-diabetic cases (FPG range: 72–123 mg/dL) and categorized them in terms of BMI into four groups. Our findings were consistent with the abovementioned studies, which found that in non-diabetic individuals, an increase in BMI is accompanied by an increase in the PPARγ expression. This increase was even remarkable in the adipose tissue of overweight subjects.

Obesity is a consequence of adipose tissue expansion. This expansion is achieved by an increase in size (hypertrophic obesity) and number (hyperplastic obesity) of adipocytes, which is considered a compensatory response to a positive balance between energy intake and energy expenditure [26]. Obesity may be accompanied by adipose tissue dysfunction, which leads to the deposition of excess fat in ectopic tissues involved in glucose homeostasis; this mechanism is the link between obesity and insulin resistance (IR) and the increased risk of type 2 diabetes [27].

In another scenario, adipose precursor cells are recruited in SAT and differentiated (healthy adipose tissue expansion). Obese individuals with an increased adipogenesis capacity in the SAT remain in a healthy metabolic state [28]. Since the persistence of a healthy metabolic state depends on increased adipogenesis in the SAT (as the largest white adipose tissue), the elevation of PPARγ expression can be explained by its role as the key regulator of adipogenesis. PPARγ, together with CCAAT/enhancer-binding protein α (C/EBPα), is known to activate the differentiation of pre-adipocytes by promoting the expression of adipogenic genes [29].

Sugii et al. showed that exclusive activation of PPARγ in adipocytes induced insulin sensitization in the body; this effect was comparable to the effect of systemic treatment with antidiabetic drugs, such as thiazolidinediones [30]. These findings are consistent with our results, which confirmed the elevation of PPARγ expression in non-diabetic obese and morbidly obese subjects. Previous studies on prediabetic cases showed a decrease in the PPARγ expression, which is an indicator of an unhealthy metabolic state or lipotoxicity [25]. Similarly, studies that had no FPG limitation and included diabetic and prediabetic individuals in the sample reported that the reduction [24] or elevation of the PPARγ gene [5] depends on the proportion of normoglycemic individuals.

Previous studies have reported the role of epigenetic modifications in the development of obesity through the regulation of PPARγ [31]. One of these underlying epigenetic mechanisms is microRNA, which is a major class of non-coding RNAs [32]. Esau et al. reported the elevated level of miR-143 in differentiating adipocytes. They also revealed that inhibition of miR-143 in transfected human pre-adipocytes inhibited adipocyte differentiation [33]. In the present study, we observed the elevation of miR-143 expression in the SAT, along with an increase in BMI, which was consistent with an increase in PPARγ expression (Fig. 1). While most of the known miRNAs have inhibitory roles, we proposed that this molecule exerts its effect on adipogenesis by inhibiting one of the process inhibitors. Zinc Finger Protein 521 (ZNF521) is a recently introduced adipogenic differentiation regulator in human cells [34]. This transcription co-factor can inhibit the expression of zinc-finger protein 423 (ZNF423) that is known to activate PPARγ transcription [35]. Although recent studies suggested that the inhibitory effect of ZNF521 is not sufficient for impaired adipogenesis and development of the unhealthy metabolic state in obesity, its repression is necessary for proceeding adipocyte differentiation [35]. Searching on the MirWalk, we found that ZNF521 mRNA is one of the predicted targets for mir-143 [36]. Inhibiting ZNF521 by mir-143 may remove its inhibitory effect on ZNF423 and activate PPARγ transcription. This regulatory mechanism can increase the expansion potency of SAT, which subsequently increases this depot’s storage capacity that could potentially prevent the development of an unhealthy metabolic state in obesity.

Several studies have shown that miR-34a plays a role in adipose tissue differentiation and is associated with obesity. Choi et al. reported the elevation of miR-34a level in the serum of obese patients compared to the controls [37]. Ortega FJ et al. also reported a positive association between the expression of miR-34a and BMI [10]. Besides, the results of a study by Shamsi et al. showed that knockdown of miR-34a in diet-induced obese mice helped them remain in a healthy metabolic state by promoting the browning of white fat depots in different sites; this effect may be exerted through suppression of PPARγ transcriptional activity [38].

In the present study, there was a significant elevation in the miR-34a expression in the VAT of obese subjects compared to normal-weight, overweight, and morbidly obese groups. On the other hand, in obese subjects, a negative association was observed between the VAT miR-34a expression and PPARγ expression. Owing to the non-diabetic state of our subjects, this observation is in accordance with the proposed inhibitory role of miR-34a in the suppression of PPARγ activity.

To the best of our knowledge, in this study, for the first time, we examined the expression of PPARγ, miR-143, and miR-34a in the human adipose tissues of four precisely defined BMI groups; this study is also the first report on an Iranian population. However, this study has some limitations that need to be underlined. First, due to the cross-sectional design of this study, causal inferences cannot be made. Therefore, future experimental studies using reporter gene assays must evaluate the proposed role of mir-143 in PPARγ transcription and adipose tissue expansion regulation. The second limitation of this study was the small sample size of the groups. Finally, since all participants in this study were from Tehran, the findings may not apply directly to other ethnic groups.

## Conclusions

The present results supported previous findings, which suggested the compensatory role of adipogenesis upregulation by preventing adipose tissue dysfunction and maintaining adequate glucose homeostasis despite obesity. The present results also confirmed the regulatory function of miR-143 and miR-34a in the PPARγ expression and adipogenesis; however, they may not be independent regulators of adipocyte differentiation and/or independent risk factors for the development of obesity.

## Data Availability

The datasets used and/or analyzed during the current study are available from the corresponding author on reasonable request.
